# Trajectories Associated With the Use and Deprescription of Benzodiazepines and Z‐Drugs in a Network of Geriatric Outpatient Clinics

**DOI:** 10.1002/pds.70419

**Published:** 2026-06-19

**Authors:** Nelson Machado do Carmo Júnior, Mariana Martins Gonzaga do Nascimento, Antônio Ignácio de Loyola Filho, Daniela Castelo Azevedo, Estevão Alves Valle, Edna Afonso Reis

**Affiliations:** ^1^ Programa de Pós‐graduação em Medicamentos e Assistência Farmacêutica, Faculdade de Farmácia Universidade Federal de Minas Gerais Belo Horizonte Minas Gerais Brazil; ^2^ Clínica Mais 60 Saúde Belo Horizonte Minas Gerais Brazil; ^3^ Departamento de Produtos Farmacêuticos, Faculdade de Farmácia Universidade Federal de Minas Gerais Belo Horizonte Minas Gerais Brazil; ^4^ Escola de Enfermagem Universidade Federal de Minas Gerais Belo Horizonte Minas Gerais Brazil; ^5^ Departamento Do Aparelho Locomotor, Faculdade de Medicina Belo Horizonte Minas Gerais Brazil; ^6^ Departamento de Estatística Instituto de Ciências Exatas, Universidade Federal de Minas Gerais Belo Horizonte Minas Gerais Brazil

**Keywords:** aged, benzodiazepines, hypnotics and sedatives, longitudinal studies

## Abstract

**Purpose:**

To determine the trajectories of benzodiazepine (BZD) and Z‐drug use among older adults (*n* = 3590) followed in a network of geriatric outpatient clinics in Brazil.

**Methods:**

This longitudinal study evaluated BZD and Z‐drug use over a 24‐month follow‐up period. The proportion of use at baseline and at the end of the study period was compared using the McNemar test. Two trajectories were considered: *started using* and *stopped using*. Comparisons between trajectories and independent variables were performed using Pearson's chi‐square test. Variables with *p* < 0.20 were eligible for multivariate analysis. Multivariate models were also used to identify factors associated with BZD or Z‐drug use at baseline and with trajectories of initiation and discontinuation. Odds ratios (OR) with 95% confidence intervals were estimated.

**Results:**

A small but significant reduction in overall BZD or Z‐drug use was observed (17.4% to 16.0%; *p* = 0.007), with a marked decrease in isolated Z‐drug use (6.1% to 4.0%; *p* < 0.001). At baseline, use was associated with female sex (OR = 1.48; 95% CI 1.19–1.84), age 60–74 years (OR = 1.64; 95% CI 1.29–2.10), having ≥ 2 fall‐related conditions (OR = 2.53; 95% CI 1.94–3.31), higher frailty scores, and cognitive dysfunction (OR = 1.32; 95% CI 1.00–1.74). Initiation was associated with pharmacist consultations during follow‐up (OR = 5.01; 95% CI 2.88–8.71), depression (OR = 2.59; 95% CI 1.80–3.73), insomnia (OR = 1.97; 95% CI 1.19–3.25), and panic syndrome. Pharmacist consultation was also associated with discontinuation (OR = 0.34; 95% CI 0.15–0.76).

**Conclusions:**

Clinical vulnerability and psychiatric conditions were associated with BZD or Z‐drug use and initiation, while pharmacist consultations were associated with both initiation and discontinuation trajectories.

## Key Points

- There was a reduction in the use of Benzodiazepines or Z‐drugs and a significant reduction in the isolated use of Z‐drugs among the older adults followed in this study.
- Female sex, younger age among older adults (60–74 years), higher frailty, cognitive dysfunction, and the presence of multiple fall‐related conditions were associated with the use of benzodiazepines or Z‐drugs at baseline.
- Depression, insomnia, and panic disorder were positively associated with the trajectory of initiation of benzodiazepine or Z‐drug use among older adults.
- Pharmacist consultations during follow‐up were associated with both initiation and discontinuation trajectories of benzodiazepine or Z‐drug use.
- The association between pharmacist consultations and medication use trajectories likely reflects greater healthcare utilization among clinically complex patients, highlighting the importance of careful interpretation of observational findings.

## Plain Language Summary

Benzodiazepines and Z‐drugs are commonly used to treat anxiety and sleep problems, but their use in older adults is associated with risks such as falls, cognitive impairment, and dependence. Understanding how the use of these medications changes over time can help to improve the care of older patients. This study followed 3590 older adults treated in a network of geriatric outpatient clinics in Brazil for 24 months. We analyzed two patterns of medication use: individuals who *started using* benzodiazepines or Z‐drugs and those who stopped using them during the follow‐up. We also examined clinical and care‐related factors associated with these changes. Overall, the use of these medications decreased slightly during the study period, with a more pronounced reduction in Z‐drug use. At the beginning of the study, the use was more common among women, aged 60–74 years, with two or more conditions related to falls, higher frailty scores, and cognitive dysfunction. Starting use during follow‐up was associated with depression, insomnia, panic syndrome, and pharmacist consultations. Pharmacist consultations were also associated with stopping these medications, suggesting that contact with healthcare professionals may influence medication use among older adults.

## 1 Introduction

Benzodiazepines (BZD) and Z‐drugs are potentially inappropriate medications (PIM) for older adults, associated with an increased risk of cognitive impairment, falls, and fractures [1, 2, 3, 4]. Pharmacokinetic and pharmacodynamic changes associated with aging increase the half‐life and effects of these drugs, raising the risk of harm [5].

However, BZD are among the most commonly used PIM by older adults in outpatient clinics worldwide [6], with a prevalence in Brazil of 4%–10% [2, 7, 8]. Prolonged use of these drugs can lead to abuse, dependence, tolerance, reduced effectiveness, immune dysregulation, and changes in the hypothalamic–pituitary–adrenal axis [9, 10, 11, 12, 13, 14].

In this sense, the deprescription of BZD and Z‐drugs is an important strategy in geriatrics to reduce adverse events, consisting of discontinuing or reducing medications that have no therapeutic benefit or cause harm [15, 16, 17, 18, 19, 20, 21]. However, it faces barriers among patients, family members, and professionals, making the process complex [22, 23, 24].

Systematic reviews with meta‐analyses indicate that deprescription of BZD and Z‐drugs in older adults is feasible, especially with non‐pharmacological support, with rates ranging from 12.3% to 43.2% in hospitals, community, long‐term care, and primary care settings [25, 26].

To the authors' knowledge, no studies have examined trajectories of BZD and Z‐drug use or related deprescription factors associated with deprescription processes or maintenance of prolonged use of these medications in older adults, particularly in geriatric outpatient clinics. In this sense, the objective of this study was to determine the trajectories of use of BZD and Z‐drugs among older adults followed in a network of geriatric outpatient clinics and the associated factors.

## 2 Methods

This longitudinal study is part of the project “Profile of medication use and deprescription in a geriatric outpatient clinic”, approved by the Research Ethics Committee of the Federal University of Minas Gerais (CAAE 52595821.1.0000.5149).

The study was conducted in a network of private geriatric clinics in Belo Horizonte, serving older adults (≥ 60 years, according to Brazilian legislation) who pay for the services or have health insurance. Patients are usually referred to by health professionals or health insurance providers when they are frail or advanced age. The clinics offer multidisciplinary care through in‐person consultations, teleconsultations, multidisciplinary meetings, and health promotion groups.

The study population consisted of all older adults admitted to the network between June 2020 and November 2024, who: (i) completed at least 24 months of follow‐up; and (ii) presented data for all variables evaluated in this study. There was only one data collection in December 2024, conducted through the generation of reports from the database of the geriatric clinic's system.

The data used in this study are secondary, coming from the outpatient clinic's health management software, called “LifeCode ‐ Inteligência & Saúde”. Data collection was blinded, through the generation of customized reports in spreadsheet file format, which were transferred to R software, version 4.3.0, where all analyses were performed.

The Anatomical Therapeutic Chemical Index (ATC Index), developed by the World Health Organization Collaboration Center for Drugs Statistic Methodology, was used to identify the drugs investigated [27]. “Benzodiazepines” refers to drugs classified under codes N05BA (e.g.,: alprazolam, bromazepam, lorazepam, diazepam) and N05CD (e.g.,: flunitrazepam, flurazepam, midazolam), in addition to clonazepam, classified as an anticonvulsant under code N03AE01, but frequently used as a sedative in Brazil. “Z‐drugs” refers to those identified with code N05CF (e.g.,: zolpidem, zopiclone and eszopiclone).

Participants were classified in relation to the investigated event into three categories: (1) isolated use of BZD, (2) isolated use of Z‐drug, and (3) use of either BZD or Z‐drug. To investigate the factors associated with the use of BZD/Z‐drug at baseline, the following variables were used: sex; age; polypharmacy (use of five or more medications) [28]; diagnosis of dementia, depression, insomnia, anxiety, or panic syndrome; number of health conditions related to falls [29, 30, 31]; number of pharmacists consultations; clinical‐functional vulnerability index (IVCF‐20) [32]; cognitive dysfunction; and change in the performance of basic activities of daily living (BADL). These last two independent variables were identified using a component of the IVCF‐20 itself.

To characterize the population at baseline, a descriptive analysis was conducted using frequencies for qualitative variables and mean, standard deviation (SD), and range for quantitative ones. The use of BZDs/Z‐drugs was analyzed from two perspectives: profile (active ingredient frequency) and prevalence of use (users vs. total participants).

The investigation of the association between the defined events (isolated use of BZD; isolated use of Z‐drug; and use of either BZD or Z‐drug) and independent variables was based on Pearson's chi‐square test (univariate analysis). Variables with *p* < 0.20 in the univariate analysis were included in multivariate logistic regression models. Associations were estimated using odds ratios (OR) and corresponding 95% confidence intervals (95% CI).

The McNemar test was performed to assess changes in the proportions of the events defined above between the beginning of the study period (6 months after admission to the network) and the end (after 24 months of connection with the network). Six months was adopted as the baseline because it is a usual period in which the patient is connected to the outpatient clinic, allowing for regular monitoring and confirmation of information regarding their health history. The Kappa coefficient was used to measure the degree of agreement between the proportions of use at the two moments (variation between 0.6 and 0.8).

Two trajectories of use of either BZD or Z‐drug were considered from the baseline to the end of the follow‐up: (1) *started using*; and (2) *stopped using*. All pharmacist consultations were conducted by telephone. Deprescribing was defined as the removal of the medication from the outpatient prescription system by either the physician or the clinical pharmacist. Similarly, medication initiation was defined as the inclusion of the medication in the prescription system. The trajectories were compared according to the independent variables using Pearson's chi‐square test (univariate analysis). Variables with *p* < 0.20 in the univariate analysis were included in multivariate logistic regression models. The stepwise automatic selection method with *p* < 0.05 was used to define the variables in the final model. Associations were estimated using odds ratios (OR) and corresponding 95% confidence intervals (95% CI).

## 3 Results

The study included 3590 older adults, the majority of whom were female (*n* = 2624; 73.1%), with a mean age of 77 years (SD = 9) (minimum = 60; maximum = 103), and a mean IVCF‐20 of 15.8 (SD = 6.9). In addition, most of the older adults had no diagnosis of dementia (90.2%), depression (73.4%), anxiety (79.9%), insomnia (87.9%), or panic disorder (98.7%) (Table 1).

**TABLE 1:** Baseline characteristics of older adults treated in a private geriatric outpatient clinic network (*n* = 3590).

Variable	Absolute frequency *n*	Relative frequency %
Sex		
Female	2624	73.1
Male	966	26.9
Age range		
60–74	1488	41.5
75–84	1318	36.7
≥ 85	784	21.8
Dementia		
No	3237	90.2
Yes	353	9.8
Depression		
No	2622	73.0
Yes	968	27.0
Anxiety		
No	2868	79.9
Yes	722	20.1
Insomnia		
No	3154	87.9
Yes	436	12.1
Panic disorder		
No	3544	98.7
Yes	46	1.3
N^o^ of health conditions related to falls		
0–1	822	22.9
≥ 2	2768	77.1
Polypharmacy^a^		
No	1761	49.1
Yes	1829	50.9
IVCF‐20^b^		
0–6	316	8.8
7–14	1457	40.9
15–40	1791	50.3
Cognitive dysfunction		
No	529	14.7
Yes	3058	85.3
Change in performance of BADL^c^		
No	3273	91.3
Yes	314	8.7

*Note:* Belo Horizonte, Minas Gerais.

^a^ Polypharmacy = use of 5 or more medications.

^b^ IVCF‐20 = Clinical and Functional Vulnerability Index.

^c^ BADL = Basic activities of daily living.

In contrast, 85.3% of the older adults had cognitive dysfunction and 77.1% had a diagnosis of two or more health conditions related to falls, although the majority (91.3%) did not demonstrate impairment in BADL. A little over half (50.9%) were using polypharmacy (Table 1).

A total of 623 older adults (17.4%) were using at least one BZD or Z‐drug. Among the 1688 BZD or Z‐drugs used by the older adults at baseline, the most frequent was clonazepam (*n* = 506; 30.0%), followed by zolpidem (*n* = 463; 27.4%). The other medications had frequencies of use below 10% (Table 2).

**TABLE 2:** Frequency of use of benzodiazepines (BZD) or Z‐drugs at baseline among older adults treated at a private geriatric outpatient clinic network (*n* = 1688).

BZD or Z‐drug medication	ATC classification system	Absolute frequency (*n*)	Relative frequency (%)
Clonazepam	N03AE01	506	30.0
Zolpidem	N05CF02	463	27.4
Alprazolam	N05BA12	396	23.5
Bromazepam	N05BA08	167	9.9
Lorazepam	N05BA06	70	4.2
Diazepam	N05BA01	30	1.8
Eszopiclone	N05CF04	21	1.2
Midazolam	N05CD08	12	0.7
Zopiclone	N05CF01	10	0.6
Flunitrazepam	N05CD03	9	0.5
Flurazepam	N05CD01	4	0.2
Total		1688	100

*Note:* Belo Horizonte, Minas Gerais.

The multivariate analysis showed that, at baseline, all investigated outcomes (isolated use of BZDs, isolated use of Z‐drugs, and use of either BZDs or Z‐drugs) were significantly associated with age (younger older adults used them more frequently) and with the number of fall‐related conditions. The outcomes “isolated use of BZDs” and “use of either BZDs or Z‐drugs” were also positively associated with female sex (*p* < 0.05), the presence of cognitive dysfunction, and the IVCF‐20 score (in all cases, *p* < 0.05) (Table 3).

**TABLE 3:** Univariate and multivariate analysis of factors associated with the use of benzodiazepines (BZD) or Z‐drugs at baseline among older individuals treated at a private geriatric outpatient clinic network (*n* = 3590).

Variable	Use of BZD		Use of Z‐drugs		Use of either BZD or Z‐drugs	
	Yes *n* (%)	OR (95% CI)	Yes *n* (%)	OR (95% CI)	Yes *n* (%)	OR (95% CI)
Sex	*p* < 0.001^a^	*p* = 0.003^b^	*p* = 0.079^a^	—	*p* < 0.001^a^	*p* < 0.001^b^
Female	346 (13.2)	1.47 (1.13;1.90)	172 (6.5)	—	502 (19.1)	1.48 (1.19; 1.84)
Male	82 (8.5)	1	48 (5.0)	—	121 (12.5)	1
Age range	*p* = 0.037^a^	*p* = 0.007^b^	*p* = 0.019^a^	*p* = 0.006^b^	*p* = 0.002^a^	*p* < 0.001^b^
60–74	195 (13.1)	1.56 (1.17; 2.08)	107 (7.2)	1.86 (1.25; 2.78)	290 (19.5)	1.64 (1.29; 2.10)
75–84	159 (12.1)	1.30 (0.97; 1.75)	80 (6.1)	1.47 (0.97; 2.23)	226 (17.1)	1.29 (1.00; 1.66)
≥ 85	74 (9.4)	1	33 (4.2)	1	107 (13.6)	1
Polypharmacy^c^	*p* = 0.533^a^	—	*p* = 0.570^a^	—	*p* = 0.573^a^	—
No	216 (12.3)	—	112 (6.4)	—	312 (17.7)	—
Yes	212 (11.6)	—	108 (5.9)	—	311 (17.0)	—
N^o^ of health conditions related to falls	*p* < 0.001^a^	*p* < 0.001^b^	*p* < 0.001^a^	*p* < 0.001^b^	*p* < 0.001^a^	*p* < 0.001^b^
0–1	46 (5.6)	1	29 (3.5)	1	71 (8.6)	1
2 or more	382 (13.8)	2.60 (1.89; 3.59)	191 (6.9)	2.12 (1.42; 3.16)	552 (19.9)	2.53 (1.94; 3.31)
IVCF‐20^d^, ^f^	*p* < 0.001^a^	*p* < 0.001^b^	*p* = 0.298^a^	—	*p* = 0.005^a^	*p* = 0.006^b^
0–6	19 (6.0)	1	17 (5.4)	—	35 (11.1)	1
07–14	161 (11.0)	1.88 (1.15; 3.09)	100 (6.9)	—	252 (17.3)	1.62 (1.11; 2.37)
15–40	246 (13.7)	2.46 (1.51; 3.99)	101 (5.6)	—	332 (18.5)	1.78 (1.22; 2.59)
Cognitive dysfunction^g^	*p* = 0.008^a^	—	*p* = 0.144^a^	—	*p* = 0.003^a^	*p* = 0.043^b^
No	45 (8.5)	—	25 (4.7)	—	68 (12.8)	1
Yes	383 (12.5)	—	195 (6.4)	—	555 (18.1)	1.32 (1.00; 1.74)
Change in the performance of BADL^e^, ^g^	*p* = 0.234^a^	—	*p* = 0.074^a^	—	*p* = 0.933^a^	—
No	384 (11.7)	—	208 (6.4)	—	569 (17.4)	—
Yes	44 (14.0)	—	12 (3.8)	—	54 (17.2)	—

*Note:* Belo Horizonte, Minas Gerais.

^a^ Pearson's chi‐square test.

^b^ Logistic regression model.

^c^ Polypharmacy: use of 5 or more medications.

^d^ IVCF‐20: Clinical and Functional Vulnerability Index.

^e^ BADL: Basic activities of daily living.

^f^ 26 older individuals with no response to IVCF‐20.

^g^ Three older individuals with no response to cognitive dysfunction and BADL.

There was an increase in the frequency of isolated use of BZD from baseline (11.9%) to the end of the follow‐up period (12.4%), but without a statistically significant difference (*p* = 0.321; Kappa = 0.7). In contrast, there was a significant reduction in the isolated use of Z‐drugs (from 6.1% to 4.0%; *p* < 0.001; Kappa = 0.63) and use of either BZD or Z‐drug (from 17.4% to 16.0%; *p* = 0.007; Kappa = 0.68) (Table 4).

**TABLE 4:** Frequency of use of benzodiazepine (BZD) or Z‐drugs among older adults treated at a private geriatric outpatient clinic network at baseline (6 months after admission) and at the end of the follow‐up period (24 months) (*n* = 3590).

Variable	Baseline *n* (%)	End of the follow‐up period *n* (%)	*p* ^a^	Kappa^b^
Isolated use of BZD			0.321	0.7
No	3162 (88.1)	3146 (87.6)		
Yes	428 (11.9)	444 (12.4)		
Isolated use of Z‐drugs			< 0.001	0.63
No	3370 (93.9)	3445 (96.0)		
Yes	220 (6.1)	145 (4.0)		
Use of both BZD or Z‐drugs			0.007	0.68
No	2967 (82.6)	3016 (84.0)		
Yes	623 (17.4)	574 (16.0)		

*Note:* Belo Horizonte, Minas Gerais.

^a^ McNemar test.

^b^ Kappa coefficient.

In Table 5, comparisons of the trajectories of either BZD or Z‐drug use are presented. The multivariate analysis showed that regarding the *started using* trajectory, having a pharmacist consultation within 24 months (OR = 5.01 [2.88–8.71]; *p* < 0.001), depression (OR = 2.59 [1.80–3.73]; *p* < 0.001), insomnia (OR = 1.97 [1.19–3.25]; *p* = 0.013), and panic disorder (OR = 1.97 [1.19–3.25]; *p* = 0.013) were positively associated with the initiation of these medications.

**TABLE 5:** Multivariate analyses with comparisons between trajectories of benzodiazepine and/or Z‐drug use among older individuals treated in a private geriatric outpatient clinic network according to independent variables (*n* = 3590).

Variable	Started using (*n* = 2967)		Stopped using (*n* = 623)	
	Yes *n* (%)	OR (95% CI)	Yes *n* (%)	OR (95% CI)
Sex	*p* = 0.081^a^	—	*p* = 0.300^a^	—
Female	104 (4.9)	—	142 (28.3)	—
Male	29 (3.4)	—	40 (33.1)	—
Age range	*p* = 0.957^a^	—	*p* = 0.193^a^	—
60–74	54 (4.5)	—	81 (27.9)	—
75–84	50 (4.6)	—	62 (27.4)	—
≥ 85	29 (4.3)	—	39 (36.4)	—
Polypharmacy^c^	*p* = 0.283^a^	—	*p* = 0.979^a^	—
No	71 (4.9)	—	91 (29.2)	—
Yes	62 (4.1)	—	91 (29.3)	—
N^o^ of pharmacists consultations in 6 months	*p* = 0.031^a^	—	*p* = 0.049^a^	—
0	124 (4.3)	—	173 (28.6)	—
≥ 1	9 (8.8)	—	9 (50.0)	—
N^o^ of pharmacists consultations in 24 months	*p* < 0.001^a^	*p* < 0.001^b^	*p* = 0.022^a^	*p* = 0.013^b^
0	108 (3.9)	1	164 (31.1)	1
1	19 (18.3)	5.01 (2.88; 8.71)	7 (13.2)	0.34 (0.15; 0.76)
≥ 2	6 (7.5)	2.00 (0.84; 4.74)	11 (26.2)	0.79 (0.39; 1.60)
N^o^ of health conditions related to falls	*p* = 0.174^a^	—	*p* = 0.447^a^	—
0–1	27 (3.6)	—	18 (25.3)	—
≥ 2	106 (4.8)	—	164 (29.7)	—
Dementia	*p* = 0.061^a^	—	*p* = 0.548^a^	—
No	126 (4.7)	—	164 (28.9)	—
Yes	7 (2.3)		18 (32.7)	—
Depression	*p* < 0.001^a^	*p* < 0.001^b^	*p* = 0.656^a^	—
No	74 (3.2)	1	93 (28.4)	—
Yes	59 (8.8)	2.59 (1.80; 3.73)	89 (30.1)	—
Anxiety	*p* < 0.001^a^	—	*p* = 0.641^a^	—
No	96 (3.90)	—	122 (29.8)	—
Yes	37 (7.28)	—	60 (28.1)	—
Insomnia	*p* < 0.001^a^	*p* = 0.013^b^	*p* = 0.973^a^	—
No	111 (4.1)	1	122 (29.3)	—
Yes	22 (9.6)	1.97 (1.19; 3.25)	60 (29.1)	—
Panic syndrome	*p* < 0.001^a^	*p* = 0.001^b^	*p* = 0.168^a^	—
No	126 (4.3)	1	178 (29.7)	—
Yes	7 (31.8)	6.52 (2.46; 17.3)	4 (16.7)	—
IVCF‐20^d^, ^f^	*p* = 0.540^a^	—	*p* = 0.550^a^	—
0–6	12 (4.3)	—	13 (37.1)	—
07–14	48 (4.0)	—	71 (28.2)	—
15–40	71 (4.9)	—	97 (29.2)	—
Cognitive dysfunction^g^	*p* = 0.867^a^	—	*p* = 0.807^a^	—
No	20 (4.3)	—	19 (27.9)	—
Yes	113 (4.5)	—	163 (29.4)	—
Change in the performance of BADL^e^	*p* = 0.464^a^	—	*p* = 0.385^a^	—
No	119 (4.4)	—	169 (29.7)	—
Yes	14 (5.4)	—	13 (24.1)	—

*Note:* Belo Horizonte, Minas Gerais.

^a^ Pearson's chi‐square test.

^b^ Logistic regression model.

^c^ Polypharmacy: use of 5 or more medications.

^d^ IVCF‐20: Clinical and Functional Vulnerability Index.

^e^ BADL: Basic activities of daily living.

^f^ 26 older individuals with no response to IVCF‐20.

^g^ 3 older individuals with no response to cognitive dysfunction and BADL.

The only variable that showed a statistically significant association with the *Stopped using* trajectory in the adjusted model was having a pharmacist consultation within 24 months (OR = 0.34 [0.15–0.76], *p* = 0.013).

## 4 Discussion

This is the first study to analyze trajectories in the use of BZD and/or Z‐drugs in a network of specialized outpatient clinics for older persons, assessing initiation or discontinuation of use over time. The results show high vulnerability among the older adults, including high mean IVCF‐20 scores, cognitive dysfunction, and conditions related to falls. There was a reduction in the use of BZD/Z‐drugs over 2 years.

The prevalence of BZD/Z‐drug use at baseline in the present study (17.4%) was higher than that identified in two other geriatric outpatient clinics (6% and 10%) [7, 33], and in a psychogeriatric outpatient clinic (13.3%) in the state of Rio Grande do Sul, Brazil [34]. But lower than the 41% observed in an outpatient clinic in the Netherlands [35]. A systematic review found that BZD and Z‐drugs are widely used by older adults in outpatient care (8.8%–82.8%) [6]. The prevalence in the present study was lower than in most studies in the review, although none of them were conducted in geriatric outpatient clinics.

The BZDs or Z‐drugs most commonly used by older adults in the study were clonazepam, zolpidem, and alprazolam. Only Cuentro et al. [8] and Carmo‐Junior et al. [2] investigated the use of these drugs in geriatric outpatient clinics in Brazil, with results similar to those of the present study, reflecting the main BZDs and Z‐drugs used in the country [36, 37]. Globally, there is a variation in the types of these medications used in outpatient clinics, with alprazolam, estazolam, clonazepam, and zolpidem being the most prevalent [38, 39, 40, 41, 42].

Compared to other BZDs, clonazepam is inexpensive, and it is relatively easy to obtain and use in Brazil, since it is available in the form of drops or tablets and is widely found in public and private pharmacies in Brazil. This could explain its prominence as the most used BZD in this study [43].

Zolpidem was one of the most widely marketed psychotropic drugs, with global production rising from 38.2 tons in 2021 to 39.1 tons in 2022, with Brazil manufacturing 4.2 tons [44, 45]. In 2024, Brazilian legislation intensified control over Z‐drugs, with the aim of systematically monitoring consumption [46]. It should be noted that extensive literature currently demonstrates that the risks to which older adults are subjected when using BZDs/Z‐drugs are equivalent, justifying the need to implement deprescription strategies for both groups of drugs [47, 48, 49].

A lower proportion of the three events was observed with increasing age, and this difference was statistically significant in the multivariate analysis. This association may reflect closer monitoring of older adults by specialized services, which favors BZD and Z‐drug deprescription [50, 51, 52]. In addition, older persons have often experienced adverse events related to BZD/Z‐drugs, which may favor the acceptance of their discontinuation [53, 54]. Our findings differ from a retrospective longitudinal study in Germany on prolonged use of benzodiazepines in the older adults, which showed an increase in therapy for more than 6 months with age (65–70 years: 12.3%; 71–80: 15.5%; 81–90: 23.7%; > 90: 31.6%), possibly due to the clinical profile and setting [55]. Differences also arise in a longitudinal study in Hong Kong, where the incidence of BZD and Z‐drug prescriptions to older persons (≥ 65 years) was the highest among all age groups [56].

Having two or more fall‐related morbidities was strongly associated with increased isolated use of BZD, isolated use of Z‐drug, or use of either BZD or Z‐drug. In other words, even considering the risks associated with falls inherent to their clinical diagnoses, older adults were using medications that can increase the occurrence of falls [57, 58, 59]. This highlights the need to raise awareness to both medication risks and harmful cofactors [2, 4, 13, 36, 49, 57, 60].

In the present study, the frequency of isolated use of BZD among older women was higher than that among older men (13.2% vs. 8.5%), a finding similar to that identified in a geriatric outpatient clinic in the state of Rio Grande do Sul (14.2% vs. 5.4%) [33]. The multivariate analysis demonstrated that female sex was associated with a 47% higher likelihood of BZD use and a 48% higher likelihood of the use of either BZDs or Z‐drugs. Additionally, the frequency of general use of the drugs quantified in the present study was also higher among older women (19.1% vs. 12.5%); this result being a differential in relation to the study cited. Women's higher anxiety prevalence stems from social and work burdens, increasing lifelong vulnerability and requiring tailored strategies [60].

The use of either BZD or Z‐drugs was independently associated with female sex, younger‐old age, fall‐related conditions, and higher frailty scores. Notably, individuals with markers of vulnerability (such as multiple fall‐related conditions and cognitive dysfunction) were more likely to use these medications, highlighting a clinically relevant paradox given their known risk profile in older adults.

Lee et al. identified that 28.1% of the elderly had psychiatric disorders, especially dementia (10.9%) and depression (9.9%), prevalences different from those observed in the present study (9.8% and 27%). In addition, they demonstrated an increase in the prescription of these drugs throughout the study period (3.44; *p* < 0.0001). The analysis revealed a decline in the use of BZD or Z‐drugs among individuals aged 65 years or older from 2021 to 2023 (−2.24; *p* = 0.0004) [56].

The slight increase in the isolated use of BZD (from 11.9% to 12.4%) observed during the period of the present study was not statistically significant. On the other hand, the small reduction in the prevalence of isolated use of Z‐drugs (from 6.1% to 4.0%; *p* < 0.001) and of use of either BZD or Z‐drugs (from 17.4% to 16.0%; *p* = 0.007) was statistically significant, drawing differences with in relation to the study by Lee et al. [56].

The geriatric outpatient clinic network in study has a systematic process for assessing patients using BZDs or Z‐drugs by a multidisciplinary health team, with aims of reducing the usage of these drugs. The results indicate that, even with specialized staff and educational initiatives, barriers to the deprescribing of BZDs and Z‐drugs persist. Literature points to factors such as dependence, patient beliefs, fragmentation of care, and the actions of other specialists [24, 61]. Future studies should explore these issues in greater depth in the context of a geriatric outpatient clinic with its peculiarities and challenges involved in dealing with a more frail and aging population.

The Clinic stands out nationally for its specialized work in providing comprehensive and interdisciplinary care to older adults, in alignment with the Brazilian epidemiological profile characterized by an aging population, multimorbidity, and polypharmacy. Nevertheless, we emphasize that the results are limited to this setting and can not be extrapolated to other contexts.

This study used trajectory modeling to understand the factors that influence the use of BZD/Z‐drugs among older adults followed in a network of geriatric outpatient clinics. No studies with a similar analysis methodology were identified that evaluated the use of BZDs or Z‐drugs in this specific setting. This highlights the novelty of the approach, especially considering the long longitudinal period adopted (24 months) and the monitoring context (network of geriatric outpatient clinics).

For the BZD/Z‐drug *started using* trajectory, the diagnoses of anxiety, depression, insomnia, and panic disorder were positively associated factors (*p* < 0.005). BZD/Z‐drug use was linked to depression in Brazilian and European older adults, often misused for insomnia or anxiety despite lacking evidence for long‐term benefit, except in panic syndrome [1, 33, 62, 63, 64].

In multivariate analyses, at least one consultation with a pharmacist in 24 months was positively associated with the trajectory *started using*. This association may reflect a broader pattern described in the literature, in which increased contact with healthcare services is linked to benzodiazepines prescribing [37]. Our results may indicate that engagement with pharmaceutical care may be demanded as a part of a healthcare trajectory that increases exposure to these medications. This relationship likely does not represent a direct causal effect of pharmacist consultations, but rather a marker of greater clinical complexity, symptom burden, or healthcare utilization.

Previous studies have shown that greater clinical complexity may hinder deprescribing efforts, as it is frequently associated with increased demand for healthcare professional involvement, patient‐level factors such as fear of symptom recurrence, including concerns about not being able to sleep, as well as resistance from patients and their families, which may further necessitate repeated consultations and ongoing professional support [64, 65, 66].

After 2 years of follow‐up, the only variable negatively associated with the *stopped using* trajectory was having consultations with pharmacists, indicating the demand for longitudinal monitoring among patients that are persistently using BZD or Z‐drugs.

These associations highlight the local demand for pharmaceutical work on safe medication use in geriatrics. In this scenario, the pharmacist reviews pharmacotherapy, identifies PIM, and provides guidance on deprescribing BZD/Z‐drugs. However, being limited to one pharmacist and the support of a pharmacy student may have reduced its impact given patient complexity.

Pharmacist consultations were associated with both *started using* and *stopped using* trajectories. This finding should be interpreted in light of the organization of pharmaceutical care in the outpatient clinics studied. In the studied scenario, if an older adult *started* using benzodiazepines or Z‐drugs, they may have been referred for pharmacist consultations, which may partly explain the association with the *started using* trajectory. At the same time, these consultations may act as a marker of the service's recognition of the importance of deprescribing and of the growing understanding among healthcare professionals about the risks of these medications in older adults.

In this context, pharmacist consultations may reflect greater healthcare utilization among patients with more complex clinical conditions. In this geriatric outpatient setting, patients with higher clinical complexity and difficulties in pharmacotherapy management, regardless of benzodiazepine or Z‐drug use, are referred to the clinical pharmacist. These referrals create opportunities for comprehensive medication review and for the reduction of potentially inappropriate medications in older adults.

The multidisciplinary care setting of the outpatient clinics may have facilitated the deprescribing process. Collaboration among physicians, pharmacists, and other healthcare professionals can enhance discussions about medication safety in older adults and support the identification and withdrawal of potentially inappropriate medications, such as benzodiazepines and Z‐drugs. A systematic review reinforces that the lack of interprofessional collaboration may be a barrier to the deprescribing process [67].

Given the originality of the present results, direct comparability with those of other studies is impossible, but a systematic review with meta‐analysis on the contribution of pharmacists to BZD deprescription among older adults in different outpatient settings demonstrated a positive impact. The review observed a tendency to interrupt or reduce the dose, especially with educational strategies and review of pharmacotherapy—findings in line with the present research [68].

Isomura et al. identified four trajectories of benzodiazepine and Z‐drug use in the Swedish population: “discontinued,” “reduction,” “slow reduction,” and “maintained” [69]. Among older adults (≥ 65 years), the “maintained” trajectory was most prevalent (36.7%). Factors associated with the reduction, slow reduction, and maintenance trajectories included initiation of treatment in primary care, initial use of hypnotics/sedatives, Z‐drugs, or multiple BZDs, and dispensing of other medications. Somatic multimorbidity increased the chance of “reduction” but reduced that of “slow reduction” and “maintenance” [69].

Although the study by Isomura et al. addresses trajectories of BZD and Z‐drug use [69], there are important differences with the present study, such as the setting in which the study was conducted and the variables associated with the trajectories, notably pharmacists' consultations, which were only addressed in the present study.

This study has limitations related to the use of secondary data, its focus on a private healthcare setting, and the lack of analysis of factors hindering medication withdrawal. The system did not capture partial dose reductions or structured information on prescribing indications, nor did it allow for the assessment of safety outcomes or withdrawal‐related adverse events. Pharmacist consultations lacked standardized documentation of their qualitative content. Additionally, the study did not evaluate pharmacist‐led interventions, nor the average dose or duration of benzodiazepine use due to system limitations that do not record these data.

Despite its limitations, the study showed that pharmacist consultation is associated with the trajectories of BZD or Z‐drug use, highlighting the local recognition of the relevance of clinical pharmacy in geriatrics. However, the findings reinforce the need to intensify deprescribing, especially in older adults with comorbidities linked to falls.

## 5 Conclusion

The present study identified clinical and care‐related factors associated with the use and trajectories of benzodiazepines and Z‐drugs among older adults followed in a network of geriatric outpatient clinics. At baseline, use was associated with female sex, younger age among older adults, greater clinical vulnerability, and the presence of fall‐related conditions and cognitive dysfunction. There was a slight reduction in the use of Z‐drugs only and in the use or either BZD or Z‐drugs.

During follow‐up, the trajectory *started using* these medications was associated with depression, insomnia, and panic syndrome, highlighting the influence of mental health conditions on prescribing patterns in geriatric care. Pharmacist consultations were associated with both *started using* and *stopped using* trajectories, suggesting the local demand for pharmaceutical care in these particular cases. This is one of the first studies in Brazil to apply trajectory analysis to investigate the longitudinal use of BZD and Z‐drugs in older adults in the context of geriatric outpatient clinics, reinforcing the need to intensify strategies for deprescribing or protecting the use of these medications.

## Funding

The authors have nothing to report.

## Disclosure

Preliminary results from this research will be presented at the ISPE LATAM Congress in Sorocaba, Brazil, in 2024.

## Conflicts of Interest

The authors declare no conflicts of interest.
